# Descending Necrotizing Mediastinitis Resulting from Pharyngitis with Perforation of the Aryepiglottic Fold

**DOI:** 10.1155/2020/4963493

**Published:** 2020-02-13

**Authors:** Alexandra Pulst-Korenberg, Stephen C. Morris

**Affiliations:** ^1^Commonwealth Health Care, Saipan (Northern Mariana Islands), USA; ^2^Department of Emergency Medicine, University of Washington, Seattle, WA, USA

## Abstract

Descending necrotizing mediastinitis and pharyngeal perforation are uncommon complications of pharyngitis that are associated with high morbidity and mortality. This case report describes a previously healthy 18-year-old male who presented to the emergency room with 5 days of severe sore throat, intermittent fevers, and vomiting and was found to have extensive posterior pharyngeal and mediastinal air along with extravasation of contrast on computed tomography, consistent with perforation of the left aryepiglottic fold as well as descending necrotizing mediastinitis. The patient had a complicated hospital course including multiple operative interventions, abscess formation, and development of pericardial and pleural effusions. Successful treatment required swift resuscitation including broad-spectrum antibiotics and significant coordination of emergent operative intervention between otolaryngology and cardiothoracic surgery. It is important to recognize descending necrotizing mediastinitis as a clinical entity that may result from oropharyngeal infections as early intervention significantly decreases subsequent complications and mortality. Furthermore, pharyngeal perforation is an extremely rare complication which requires either CT with oral contrast or esophagram for diagnosis.

## 1. Introduction

Sore throat is a common chief complaint of Emergency Department (ED) visits among young, healthy individuals, most of which result in a diagnosis of uncomplicated bacterial or viral pharyngitis [1]. Common complications of pharyngitis include retropharyngeal abscess, parapharyngeal abscess, and peritonsillar abscess. Only 2-3% of deep space neck infections progress on to more serious infections such as mediastinitis [2, 3]. The authors report here a case of a sore throat that progressed to perforation of the left aryepiglottic fold, resulting in mediastinitis.

Deep space neck infections and mediastinitis usually develop from a nidus of infection such as odontogenic, sinus, or lymphangitic in the former or violation of the deep space by iatrogenic surgical complication or traumatic event such as esophageal rupture in the latter. The pathology of deep space infection converting to mediastinitis involves the infection migrating along tissue planes and is rare in the antibiotic era. There are 3 main spaces of the cervical soft tissues: submandibular, parapharyngeal, and retropharyngeal. Posterior to the retropharyngeal space, between the prevertebral and alar fascia below the base of the skull, is the “danger space,” named so because it is the only space that freely connects to the posterior mediastinum, making it the primary route for direct spread of infection [4]. This process with resultant infection in the head, neck, and chest has been termed “descending necrotizing mediastinitis” with case reports and surgical recommendations dating from 1938 [5].

Perforation of the aryepiglottic fold is far less common than esophageal perforation, which is commonly associated with instrumentation, forceful vomiting (Boerhaave's), severe coughing, or substance abuse. As the case described here includes none of these predisposing factors, this case represents some significant unknown pathology as to why the patient developed a perforation and secondly why it progressed to severe mediastinitis. The aim of this case report is to inform clinicians so that they may recognize these severe conditions and provide guidance on early diagnosis and intervention.

## 2. Case Presentation

A healthy 18-year-old male living in a mobile home in rural Washington with his family noticed mild throat and left ear pain 5 days prior to presentation. His symptoms steadily worsened and became severe 3 days prior to admission to the point where ibuprofen 600 mg and chloraseptic spay provided only minimal relief. In the 2 days prior to admission, he developed fever to 38.4 degree Celsius, malaise, poor oral intake, nonproductive cough, dysphagia, odynophagia, and 3-4 episodes of nonbilious and nonbloody vomiting. The remainder of his history was notable only for a tethered spinal cord which was repaired as an infant, a 1–1.5 pack per day cigarette use, and no alcohol or illicit drug use. On presentation to the local rural hospital, he was noted to be febrile and tachycardic, though handling his secretions adequately, and was nontoxic appearing.

After laboratory analysis revealed a leukocytosis of 26,000, raising the initial concern for a more serious infection, a CT scan of his neck with IV contrast was obtained, revealing “extensive air in the parapharyngeal and retropharyngeal space extending to the supraclavicular region and mediastinum.” He was treated with IV fluids, an antipyretic, morphine, clindamycin 900 mg IV, and dexamethasone 4000 mg IV and was transferred to a tertiary care center for a higher level of care and specialty consultation. He had improved hemodynamics after these interventions, with a heart rate of 79, blood pressure of 111/69, and a temperature of 37.7 degrees Celsius.

On arrival to the tertiary care center, he was managing his secretions, reporting some persistent throat discomfort, and intermittently spitting up saliva with small flecks of blood for purposes of comfort. Physical exam was notable for absence of distress, mild tenderness, and erythema to anterior neck left greater than right, poor dentition, symmetrical tonsillar swelling with no uvular deviation, and pharyngeal erythema. Given lack of stridor, shortness of breath, or inability to tolerate secretions, there was no immediate concern for airway compromise.

A repeat CT scan of the neck soft tissues with IV and oral contrast obtained approximately 7 hours after the initial scan demonstrated extensive fluid and gas collections within the parapharyngeal and retropharyngeal spaces extending from the skull base to the anterior mediastinum that were radiographically concerning for necrotizing soft tissue infection, as well as a small amount of contrast extravasation at the left aryepiglottic fold/pyriform sinus, concerning for perforation and communication with the retropharyngeal fluid and gas collections.

Operative culture of the retropharyngeal fluid collection eventually grew viridans streptococci, *Candida dubliniensis*, alpha hemolytic streptococci, yeast, in addition to mixed anaerobic flora and Gram-positive cocci unable to be further speciated.

Upon viewing the initial CT scan, the care team, including the otolaryngology and thoracic surgery team, felt the patient had retropharyngeal cellulitis with soft tissue gas/air extending to the mediastinum, the differential diagnosis of which included primary cellulitis, mediastinitis, and perforation of unclear etiology with a possible odontogenic source.

The patient's overall presentation, with initially compromised hemodynamics and systemic signs and symptoms of infection (tachycardia, fever, leukocytosis, and oropharyngeal erythema/oedema), was clearly concerning for more than a simple viral or streptococcal pharyngitis and is highly suggestive of sepsis. The second CT which included both oral and IV contrast was critical in pointing toward a far more serious infection than is typically associated with retro- and parapharyngeal abscesses. The presence of multiple air and fluid collections was radiographically concerning for necrotizing soft tissue infection including the mediastinum, rather than a simple cellulitis of the soft tissues of the neck and oropharynx. This CT also pointed toward a possible source, based on the contrast extravasation indicative of perforation. Esophageal perforation, mucosal ulceration or preexisting inflammation, and malignancy predisposing the patient to perforation also should be included in the differential for any patient with unexplained oropharyngeal perforation, though was felt to be less likely here given the imaging findings, short duration of symptoms, and young age.

Otolaryngology and thoracic surgery were consulted soon after arrival to the ED for admission and likely operative intervention. The patient was admitted to the surgical intensive care unit for continued resuscitation and carefully watched on possible impending airway compromise due to the amount of inflammation potentially leading to significant airway edema. Antibiotic coverage was broadened from the initial clindamycin to 2 grams of vancomycin IV and 4.5 grams of piperacillin-tazobactam IV based on the concern for sepsis, perforation, and extensive air concerning for mediastinitis and necrotizing soft tissue infection. He was also instructed to have nothing by mouth, given maintenance fluids in anticipation of operative management, and his pain was controlled with IV hydromorphone. The initial management plan also involved a plan for an esophagram to evaluate the location of perforation and possibility of multiple perforations, which would change operative management. In the morning of hospital day 2, a third CT scan revealed marked expansion of the gas and fluid within the soft tissues of the parapharyngeal, retropharyngeal, and mediastinal spaces. The patient was therefore taken to the operating room, and a transoral drainage of his retropharyngeal abscess was conducted via open incision by otolaryngology with culture obtained.

The patient initially improved, but on hospital day 4, he began complaining of chest pain and reduced neck range of motion, with exam notable for tachycardia and blanching erythema on his chest. His white blood cell count had increased over the preceding days from 24,000 to 44,000/microL. A repeat CT of the chest with IV contrast demonstrated bilateral pleural effusions and pericardial effusion, both of which are known complications of mediastinitis. The patient was then taken to the operating room by thoracic surgery, who placed bilateral thoracostomy tubes and a mediastinal Jackson–Pratt drain, with extensive purulent material drained. Transcervical drainage and washout of recurrent retropharyngeal abscess was performed during the same operative period by otolaryngology. Ultimately, operative management required multiple washouts of the retropharyngeal abscess cavity, multiple right thoracotomy washouts, and a pericardial window.

The patient received twice daily washouts for 5 days. By hospital day 14, the thoracostomy tubes and Jackson–Pratt drain were removed. He was further evaluated by dentistry who noted heavy accumulation of plaque and poor dental hygiene with gingivitis but did not identify any intervenable oral pathology or clear nidus of odontogenic infection.

The patient was transitioned to oral antibiotics and discharged on hospital day 17 with amoxicillin/clavulanate 875 mg twice daily for 15 days postoperatively, as recommended by infectious disease, with follow-up arranged for primary care, dentistry, otolaryngology, and thoracic surgery.

## 3. Discussion

This is an unusual case of pharyngitis leading to spontaneous oropharyngeal perforation and descending necrotizing mediastinitis. Other serious complications of deep space neck infections include airway obstruction, empyema, sepsis, erosion of major blood vessels with the potential for catastrophic bleeding, and cardiac tamponade [6]. Considerations for treatment include rapid recognition of shock, early broad-spectrum antibiotic therapy, and intubation in the event of airway edema with fiberoptic laryngoscopy as the airway architecture may be distorted.

Radiographic studies such as lateral neck X-ray, posterior and lateral chest radiographs, or abdominal series have historically been part of the diagnostic workup; however, signs such as prevertebral air or space widening, subcutaneous emphysema, and mediastinal widening are subtle and potentially misleading [7]. In the age of rapidly available CT, these studies may simply delay definitive diagnosis and treatment.

Despite being a rare complication, mediastinal extension of deep space neck infections is an important complication to consider when a patient presents with toxic-appearing with pharyngitis due to the historical high mortality. A comparison of case studies and meta-analysis conducted after a literature review in 2012 demonstrated a mortality ranging from 6% to 40%, with 5 of 7 reports demonstrating mortality greater than 30% [4]. Cervical necrotizing fasciitis alone has been reported to have mortality rates ranging from 7% to 20% depending on the extent of neck soft tissue involved; when combined with descending mediastinitis, reported mortality reached up to 41%, with the presence of sepsis being the single worst prognostic factor [8]. There is a strong association of early diagnosis and treatment with improved survival of descending necrotizing mediastinitis, as well as with decreased progression of deep space neck infections to mediastinitis, highlighting the need for early recognition in front line clinicians [8, 9]. Case reports suggest that delaying surgical debridement significantly increases mortality, necessitating early suspicion to facilitate specialist consultation and transfer to a higher level of care if cardiothoracic surgery or otolaryngology is unavailable [10]. Alternative treatments such as hyperbaric oxygen and ozone therapy for mediastinitis have also demonstrated potential but are not currently the standard of care. Interestingly, the mortality of mediastinitis secondary to spread from deep space neck infections is higher than overall mortality of mediastinitis [8]. One potential explanation could be delay in diagnosis, as only 15–34% of cervical necrotizing fasciitis is accurately diagnosed at presentation [11]. Evidence also suggests that late diagnosis of oesophageal rupture (greater than 24 hours) significantly increases morbidity and mortality [12].

The location of perforation, at the aryepiglottic fold, is also remarkable. Esophageal perforation secondary to vomiting, also known as Boerhaave's, is much more common than pharyngeal perforation and typically occurs in the lower third of the esophagus at an area of natural narrowing [12]. The aryepiglottic fold, which forms the medial border of the pyriform sinus, may have an increased risk of perforation as compared to surrounding oropharyngeal tissue as the pyriform sinus lacks a longitudinal muscular layer, which reinforce the mucosal layers when faced with increased pressures such as with vomiting [12, 13]. Oral contrast extravasation should be visible on CT imaging as in this case, and in cases of suspected pharyngeal perforation, CT of the neck and thorax with water soluble contrast is the diagnostic modality of choice [14]. If esophageal perforation is suspected, a contrast esophagram remains the gold standard study, as it is the most reliable in demonstrating the presence and location of an esophageal perforation [7].

There are only a handful of other case reports of spontaneous pharyngeal perforation: one in association with necrotizing mediastinitis, odontogenic infection, and others related to forceful vomiting in the context of a “common cold,” vaginal delivery, or even sneezing against closed mouth and nostrils [12–15]. The first of these cases involved a middle-aged male with a history of both alcohol abuse and uncontrolled diabetes mellitus whose symptoms began with forceful vomiting without signs of infection, and only 1 week later developed signs of a deep space neck infection [13]. To our knowledge, the case presented here is the second case in the published literature of pharyngitis leading to spontaneous perforation and descending necrotizing mediastinitis. It is particularly remarkable that our patient was under 20 years old, and that his only risk factor was tobacco use, in contrast to the first case report, which is a more standard reflection of risk factors found in the literature. Multiple literature reviews of published cases highlight diabetes mellitus, immune suppression, alcohol abuse, and tobacco abuse as risk factors for the development of necrotizing infections of the neck and descending mediastinitis [8, 10].

In sum, clinicians must maintain a high index of suspicion when faced with cases of atypical pharyngitis as descending necrotizing mediastinitis is associated with high morbidity and mortality, and early recognition and intervention can be life-saving.

## Supplementary Materials

Supplementary Materials The supplementary materials consists of a CT scan demonstrating air tracking from pharyngeal space to the mediastinum and CT scan demonstrating mediastinal free air, pericardial and pleural effusions.

## References

[1] Hartman N. D., Tintinalli J. E., Stapczynski J., Ma O., Yealy D. M., Meckler G. D., Cline D. M. (2016). Neck and upper airway. *Tintinalli’s Emergency Medicine: A Comprehensive Study Guide*.

[2] Marioni G., Staffieri A., Parisi S. (2010). Rational diagnostic and therapeutic management of deep neck infections: analysis of 233 consecutive cases. *Annals of Otology, Rhinology & Laryngology*.

[3] Huang T.-T., Liu T.-C., Chen P.-R., Tseng F.-Y., Yeh T.-H., Chen Y.-S. (2004). Deep neck infection: analysis of 185 cases. *Head & Neck*.

[4] Kocher G. J., Hoksch B., Caversaccio M., Wiegand J., Schmid R. A. (2012). Diffuse descending necrotizing mediastinitis: surgical therapy and outcome in a single-centre series. *European Journal of Cardio-Thoracic Surgery*.

[5] Pearse H. E. (1938). Mediastinitis following cervical suppuration. *Annals of Surgery*.

[6] Wang K.-Y., Lin H.-J., Chen Y.-H. (2012). Retropharyngeal abscess with descending necrotizing mediastinitis. *The Journal of Emergency Medicine*.

[7] Kaman L., Iqbal J., Kundil B. (2011). Management of esophageal perforation in adults. *Gastroenterology Research*.

[8] Sarna T., Sengupta T., Miloro M., Kolokythas A. (2012). Cervical necrotizing fasciitis with descending mediastinitis: literature review and case report. *Journal of Oral and Maxillofacial Surgery*.

[9] De Freitas R. P., Fahy C. P., Brooker D. S. (2006). Descending necrotising mediastinitis: a safe treatment algorithm. *European Archives of Oto-Rhino-Laryngology*.

[10] Quereshy F. A., Baskin J., Barbu A. M., Zechel M. A. (2009). Report of a case of cervicothoracic necrotizing fasciitis along with a current review of reported cases. *Journal of Oral and Maxillofacial Surgery*.

[11] Suárez A., Vicente M., Tomás J. A., Floría L. M., Delhom J., Baquero M. C. (2014). Cervical necrotizing fasciitis of nonodontogenic origin: case report and review of literature. *The American Journal of Emergency Medicine*.

[12] Roh J.-L., Park C. I. (2008). Spontaneous pharyngeal perforation after forceful vomiting: the difference from classic boerhaave’s syndrome. *Clinical and Experimental Otorhinolaryngology*.

[13] Chen T.-C., Wu M.-H., Cheng Y.-J., Chang P.-C. (2011). Spontaneous pharyngoesophageal perforations. *European Journal of Cardio-Thoracic Surgery*.

[14] Yang W., Sahota R. S., Das S. (2018). Snap, crackle and pop: when sneezing leads to crackling in the neck. *BMJ Case Reports*.

[15] Fraser I. D., Williams G. T. (1975). Spontaneous rupture of the pharynx. *The Journal of Laryngology & Otology*.

