# Supplementary material for: Descending Necrotizing Mediastinitis Resulting from Pharyngitis with Perforation of the Aryepiglottic Fold
Source: Case Rep Emerg Med. 2020 Feb 13;2020:4963493. doi: 10.1155/2020/4963493 (PMC7040390; doi:10.1155/2020/4963493)
Supplement: Supplementary Materials — The supplementary materials consists of a CT scan demonstrating air tracking from pharyngeal space to the mediastinum and CT scan demonstrating mediastinal free air, pericardial and pleural effusions. [file 4963493.f1.docx]

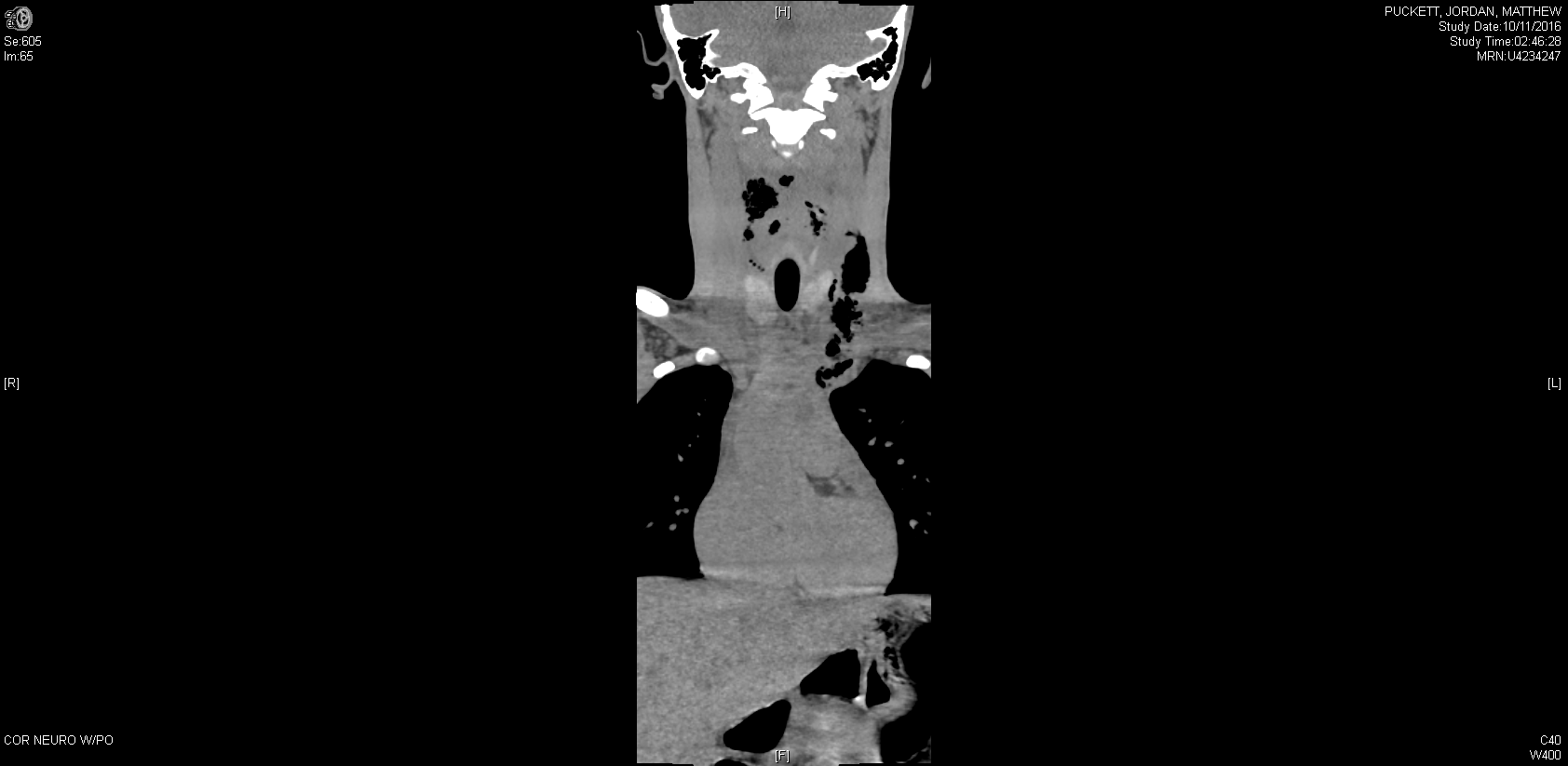


CT scan demonstrating air tracking from pharyngeal space to the mediastinum.


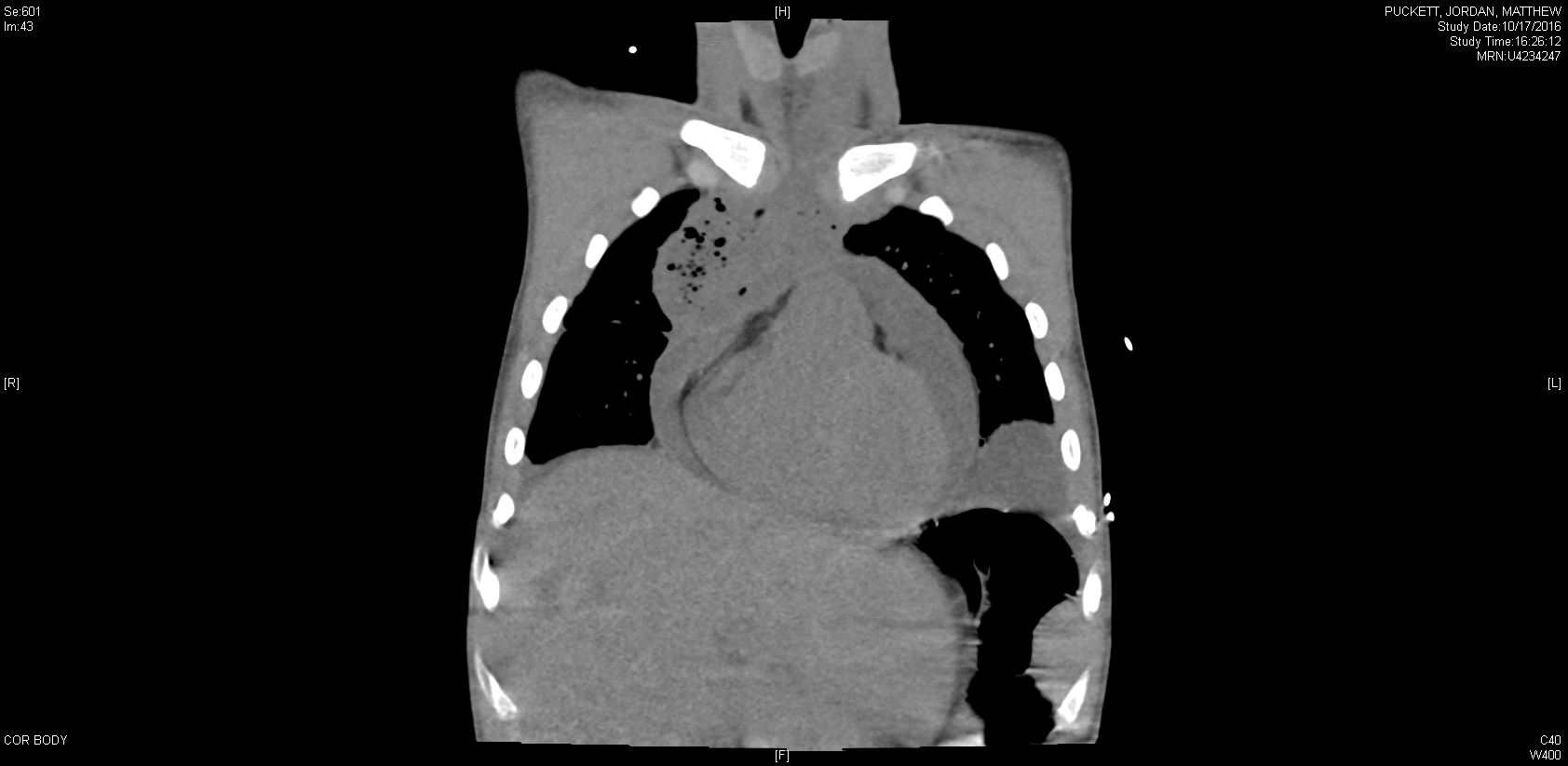


CT scan demonstrating mediastinal free air, pericardial and pleural effusions.
